# Biologically Based Restorative Management of Tooth Wear

**DOI:** 10.1155/2012/742509

**Published:** 2012-01-18

**Authors:** Martin G. D. Kelleher, Deborah I. Bomfim, Rupert S. Austin

**Affiliations:** ^1^King's College London Dental Institute, Denmark Hill, London SE5 9RT, UK; ^2^Eastman Dental Hospital, 256 Gray's Inn Road, London WC1X 8LD, UK; ^3^King's College London Dental Institute, Guy's Hospital, London Bridge, London SE1 9RT, UK

## Abstract

The prevalence and severity of tooth wear is increasing in industrialised nations. Yet, there is no high-level evidence to support or refute any therapeutic intervention. In the absence of such evidence, many currently prevailing management strategies for tooth wear may be failing in their duty of care to first and foremost improve the oral health of patients with this disease. This paper promotes biologically sound approaches to the management of tooth wear on the basis of current best evidence of the aetiology and clinical features of this disease. The relative risks and benefits of the varying approaches to managing tooth wear are discussed with reference to long-term follow-up studies. Using reference to ethical standards such as “The Daughter Test”, this paper presents case reports of patients with moderate-to-severe levels of tooth wear managed in line with these biologically sound principles.

## 1. Introduction

Tooth wear (TW), also known as tooth surface loss (TSL), is an insidious and cumulative multifactorial process involving destruction of enamel and dentine which can threaten tooth survival and the oral health related quality of life of affected individuals [1, 2]. Despite the overall trends towards improved oral health and reduced dental caries incidence over the last decades, epidemiological evidence supports the contention that TW is increasing in severity and prevalence, not only amongst older people who are living longer and retaining more teeth, but also amongst those in the early decades of their adult life [3, 4].

Greater understanding of the pathophysiology of TW has driven advances in dental materials and techniques for the benefit of affected patients. These advances have led to biologically based prosthodontic strategies that challenge many traditional or currently prevailing concepts of TW management. This is especially the case when one considers the ethical health care maxim: “Firstly, do no harm” (Primum est non nocere). Adopting biologically sensible TW management strategies will ensure that as much good as possible is achieved for the patient (beneficence) whilst avoiding harm (nonmaleficience) and upholding the patients' rights to have the reasonable treatment undertaken that most closely matches their wishes and expectations (autonomy). Traditional concepts must now be reassessed in order to achieve a radical paradigm shift in the philosophies behind TW management.

This paper will therefore, review the fundamental principles that should be considered when deciding how to manage patients with TW. The current state of knowledge of the aetiology and differential diagnoses of TW will be discussed, followed by an analysis of patient wishes and expectations when seeking sensible solutions for their TW problems. The relative risks and benefits of the possible management options will then be weighed up with reference to current available evidence. Possible solutions which aim to put patients' long-term interests first will be outlined with reference to ethically sound healthcare principles and using some case examples.

## 2. Aetiology and Differential Diagnoses of Tooth Wear (TW)

There are three main, or widely recognized, aetiologies of TW, namely, erosion, attrition, and abrasion [5]. There is a fourth aetiological factor which has been recognized by some but is certainly not universally accepted, namely, abfraction [6]. Each of these has many different clinical presentations which can be challenging to accurately diagnose, because the aetiology is usually multifactorial [7].

### 2.1. Erosion

**Figure 1:**
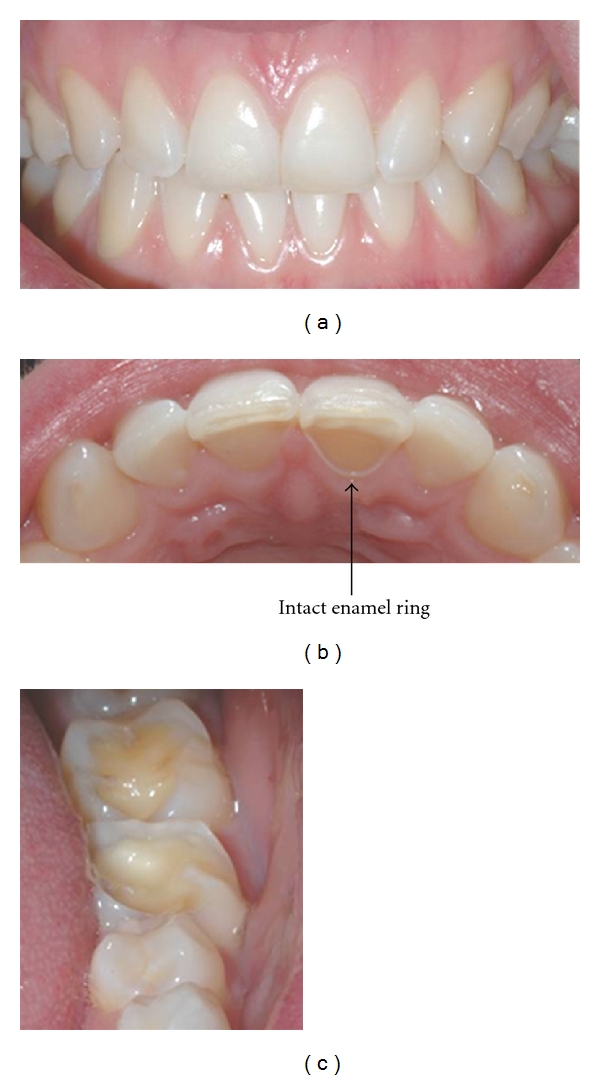
Varying severities of erosion observed in the (a) anterior maxillary facial surfaces, (b) anterior maxillary palatal surface, and (c) posterior mandibular occlusal surfaces of a 23-year-old female.

Erosion (loss of tooth tissue by the chemical dissolution of enamel or dentine by the action of nonbacterial acids from dietary or gastric sources) initially appears as “silky-glazed” dull enamel surfaces, with loss of enamel characterization such as perikymata [8]. In moderate cases, the buccal and lingual surfaces of maxillary anterior teeth appear smooth and shiny with the loss of some anatomical features (Figure 1(a)). In advanced cases, there is complete loss of the enamel, and dentine is exposed. Often an intact ring of enamel is spared and remains present around the gingival area of the teeth, as seen in Figure 1(b). This enamel ring is probably due to the neutralisation of the acid by the gingival crevicular fluid. As the lesion advances, multiple hollowed or cupped out areas form on the occlusal surfaces [9], as seen in (c) of Figure 1.

In gastric erosion, the palatal surfaces of maxillary anterior teeth are initially affected, as the tongue and the buccal mucosa protect the other surfaces from exposure to gastric acid [10]. However, as the condition progresses, the protective effect is often lost, and the erosive TW becomes more widespread [11, 12]. In contrast, dietary erosion presents as widespread cupped out lesions with the specific pattern being dependent upon the specific habits and diet of the patient. Diagnosis is critical in order to protect the remaining invaluable sound tooth structure appropriately.

The increasing prevalence of erosion in industrialized societies has complex interplaying causes. Modern sociocultural standards of the “ideal” body image have resulted in the ubiquitous use in the media and fashion industries of very slim models to portray the supposedly ideal female body shape and size. Increased exposure to such media has been shown to be psychologically detrimental to the well-being [13] and perceived self-image [14] of many young women. The emphasis on thinness may cause impressionable young people to adopt obsessive dieting and/or exercise behaviours, and there is evidence that increased exposure to such media may lead to an increased risk of eating disorders such as anorexia nervosa [15]. One unfortunate outcome of these pressures to be slim can be excessive oral exposure to both dietary and gastric acids which may partly explain the increased prevalence of TW in young adults in the UK between 1998 and 2009 [3].

**Figure 2:**
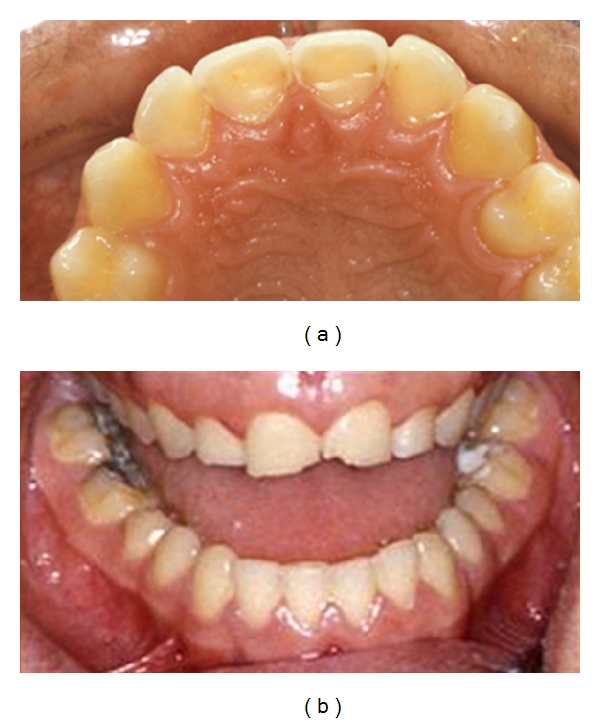
Localized tooth wear of a 15 year old.

Refrigerated transportation and increased soft drinks consumption are also likely to contribute to the increase in dietary dental erosion, as fruit and fruit juices are available all year round rather than being limited by seasons. If fresh fruits (particularly fruits containing citric or malic acid with high titratable acidity) are consumed regularly, then the frequency of acid contact episodes increase as does the risk of dental erosion [16]. The quantity and quality of saliva, salivary pellicle, physiological soft-tissue movements, and tooth anatomy and position in relation to the soft tissues will also influence the development and progression of erosive TW [17]. Behavioural factors, such as style and frequency of eating and drinking, have important consequences. An acidic drink that is held, “swished,” “swirled,” or “sluiced” in the mouth before swallowing will increase the contact time of the solution with the tooth surface and therefore the risk of dissolution of the hard tissues increases [18]. Frequent sipping of small quantities of acidic drinks will also increase their erosive potential [5].  Figure 2 shows localized tooth wear due to frequent sipping of carbonated drinks in a 15 year old. The area of erosion corresponds to the ring pull (V shaped) area of the can, which explains the localisation of the erosion to the central incisors only and the relative sparing of the lateral incisors and canines.

### 2.2. Attrition

**Figure 3:**
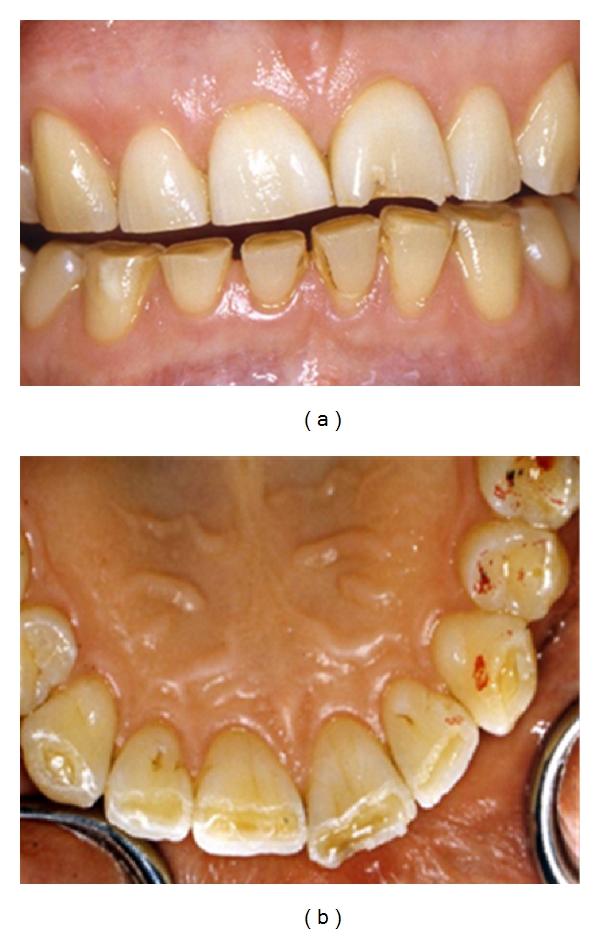
Photographs of a patient with attritional wear of the maxillary and mandibular teeth.

Attrition (wear of dental hard tissue as a result of tooth-to-tooth contact with no foreign substance intervening) usually affects the incisal/occlusal surfaces of teeth in such a way that the opposing occluding surfaces of mandibular and maxillary teeth interrelate [19]. These lesions are often flat and glossy and have distinct margins. There is usually symmetry with an antagonist tooth but this is often in one of the border positions and frequently not in their habitual intercuspal position. The specific pattern of wear coincides with how and where the patient bruxes or rubs their teeth forcibly against one another during their parafunction.  Figure 3 illustrates a pattern of attritional TW which shows even wear of the maxillary and mandibular teeth. Although there is considerable evidence of bruxism, there is a marked absence of buccocervical wear lesions, which further refutes the role of stress-related wear (abfraction) as a plausible cause of buccocervical TW.

### 2.3. Abrasion

**Figure 4:**
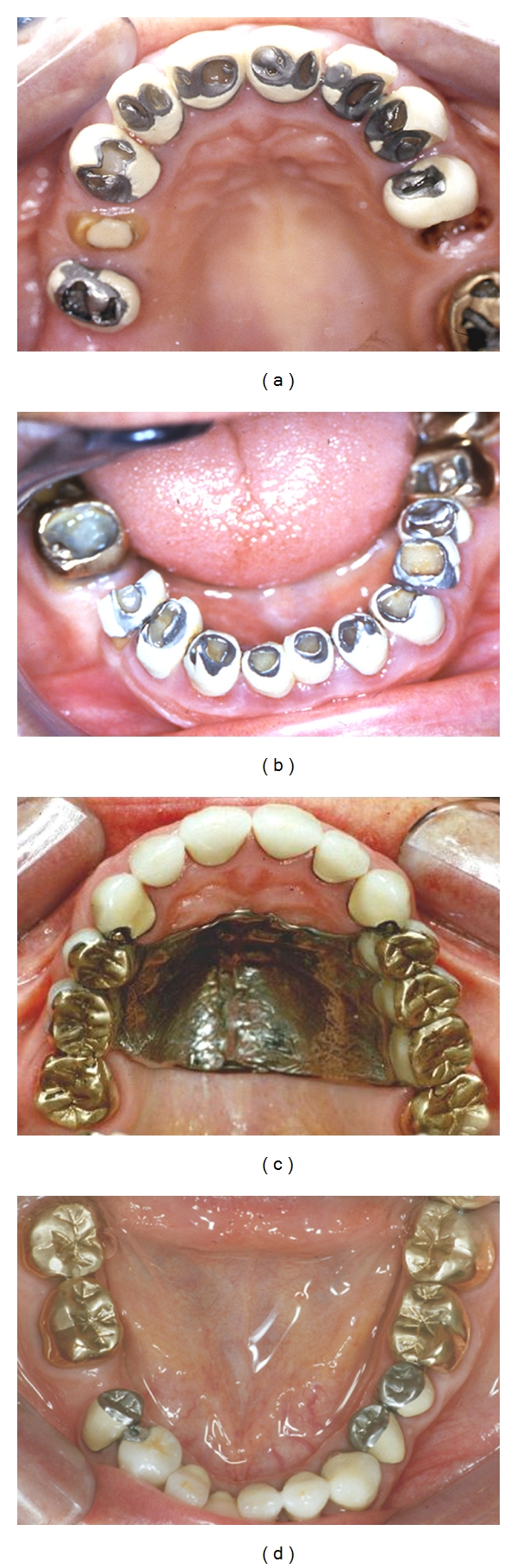
Photographs of (a, b) extensively abraded crowns and (c, d) the subsequent treatment.

Abrasion (mechanical wear of dental hard tissue not involving tooth-to-tooth contact) often presents in the cervical region of teeth, especially when associated with tooth brushing habits removing acid softened enamel and dentine in areas where gingival recession has occurred [19]. Other causes of abrasion include patients chewing abrasive materials such as sand.  Figure 4 shows photographs of a patient who chewed sand as a habit. Figures 4(a) and 4(b) show the extensive abrasion of her third full mouth reconstruction in five years. The patient was referred for psychiatric help with her destructive habits. As the dentition has previously been extensively prepared for conventional porcelain crowns, the dentition was subsequently restored, as shown in (c)-(d), using conventional materials and metal on occluding surfaces.

## 3. Principles of Management

### 3.1. The Importance of Differential Diagnosis

Dietary, medical, social, and dental histories are very important to help to distinguish between the various clinical presentations. It is especially important to distinguish erosion from attrition so that the TW can be managed appropriately, taking into account the very different aetiologies and their consequences [20]. For instance, if the chemical dissolution from dietary acids is the main aetiological factor, then the restorative material primarily needs resistance to acid attack in order to protect the remaining sound tooth tissue from further acid dissolution.

Once the aetiology has been investigated in detail, a “best fit” diagnosis has been made and the patient concerns have been determined, the main aims of biologically sensible management are the following:

1. the preservation of the remaining tooth tissue,
2. a pragmatic improvement in aesthetics,
3. the restoration of patient confidence (both in terms of their ability to manage their own condition and the likelihood of their remaining tooth tissue lasting for the rest of their lifetime).

The preservation of tooth structure is of the greatest concern. In cases of gastric erosion, it is of real importance to prevent further exposure of the eroded teeth to the damaging gastric contents [21]. The management strategy may include, for example, referring the patient to a gastroenterologist for medical management of their gastro-oesophageal reflux or to a psychologist or psychiatrist for behavioural and/or psychological management of an eating disorder [22]. Effectively managing gastro-oesophageal reflux has been shown to reduce enamel erosion even within a six week period of effective treatment [23]. However, with the exception of management of gastric erosion, there is, currently, no high-level evidence about the clinical effectiveness of any other preventative measures such as monitoring periods and/or the use of oral care products in TW [24].

### 3.2. Choices of Materials for Managing Tooth Wear

**Figure 5:**
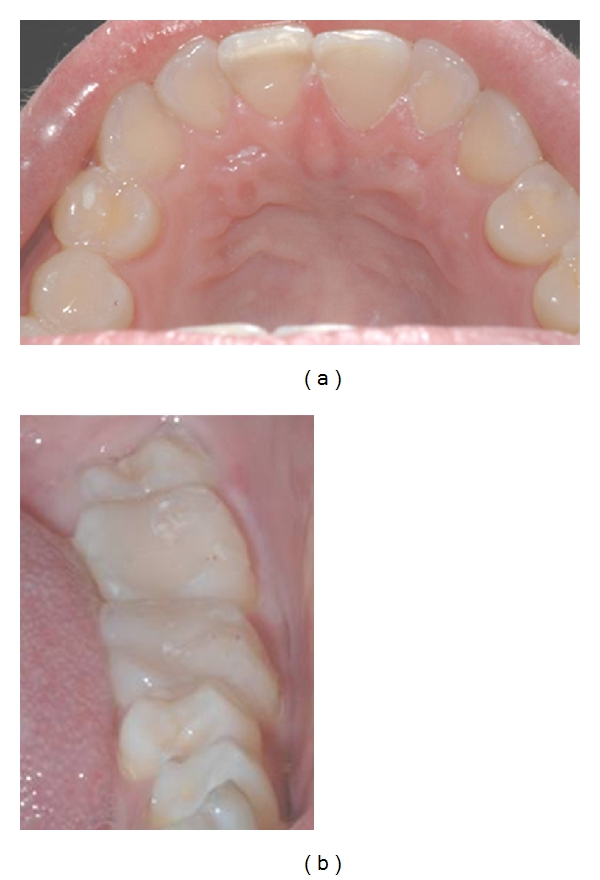
Biologically pragmatic treatment of erosion using direct resin composite in a 23-year-old female (compare to Figure 1).

Epidemiological data from industrialized countries such as the UK show that increasingly more patients will retain many of their teeth for their lifetime [3]. Therefore, any dental material used to manage TW must ensure the survival of the structural strength of the underlying remaining tooth tissue. It follows, therefore, that the survival of the tooth is of paramount importance, and by way of comparison, the survival of the restoration is of far less consequence. In fact, because modern restorative materials are now considered expendable, reparable, and renewable, the traditional full mouth rehabilitation approach as a rationale for restoring a worn dentition must now change and focus instead on protecting the remaining sound tooth structure. Studies into the use of dentine bonding agents as a management strategy have found that the coating was retained for a short period of time only [25].  Figure 5 shows an example of directly applied resin composite bonded at an increased vertical dimension in order to provide long-term protection to (a) the eroded palatal surface of a maxillary incisor and (b) the occlusal surfaces of mandibular molars (compare with Figure 1).

### 3.3. Patients' Wishes and Expectations

The social and psychological impact of dental disease have been well documented, and it has been shown that poor oral health has a detrimental effect on one's ability to live comfortably, be successful in employment, enjoy life, experience relationships, and possess a positive self-image [26]. When seeking a solution for the management of patients with TW, it is important to determine what specific aspects of the problems are of most concern to the patient, for example, lack of visibility or sharpness of teeth, sensitivity to thermal changes, or the colour of their teeth or shape problems.

This brings patient's wishes and expectations to the forefront by considering how they wish to have their problem managed. When the biologically sound concept of protecting their remaining sound tooth tissue is explained to patients, not only do they readily understand the value and rationale of such an approach, but also they usually actively seek to avoid destructive treatments which would remove more of their sound tooth tissue, and involve possible pulpal damage, loss of vitality, further endodontic complications, or ultimately loss of aggressively treated teeth [27]. It is vital to remember that the survival of the tooth and dentinopulpal complex is of paramount importance, and therefore, the focus should be on the survival of the tooth or teeth rather than on the success or survival of the restorations. This biologically sensible approach accepts a lifetime of repair and renewal of restorations, in lieu of, further loss of sound tooth tissue. 

When choosing the appropriate material to manage TW, “The Daughter Test” in elective aesthetic dentistry is of pertinence [28]. This test proposes that whenever elective intervention is contemplated, the following question should be asked: “Knowing what I know about dentistry and the effects of this elective treatment on the health and structure of these teeth in the long-term, would I carry out this treatment on my own daughter?” [28]. If, in answering this question honestly, dentists would be unwilling to carry out electively destructive treatment on their own daughter (or son, younger sister/brother, mother/father, husband/wife), then why would they ever consider carrying out such dental treatment on one of their trusting patients?

There is evidence that when patients are adequately informed, most prefer more conservative options such as resin composite rather than destructive options such as porcelain. Patients do not perceive porcelain restorations to be necessarily more aesthetic than resin composite restorations [27]. Using resin composite to manage TW is conservative, predictable, and usually aesthetically acceptable to patients (Figure 5). The use of resin composite is also safe with minimal long-term pulpal or structural complications being reported when this is applied to the external aspects of teeth in thick section. Long-term follow-up studies show that the main complications are repairable and retrievable with no loss of tooth vitality or need for loss of further teeth [29].

In marked contrast, the use of dental porcelain veneered onto various copings requires much more of the remaining sound tooth tissue to be removed from the already worn teeth in order to gain adequate space for the brittle porcelain. Few patients with tooth wear are told that in order to have an all-ceramic or ceramic-bonded-to-metal approach to manage their wear that much more of their already reduced teeth will be further destroyed. Edelhoff and Sorensen [30] demonstrated that when teeth are prepared for metal-ceramic or all-ceramic crown between 63%–72% of coronal tooth structure is removed. The long-term biological costs of this amount of elective structural and pulpal damage can be potentially huge. Ethical clinicians are under a duty of care to ensure that informed consent has been obtained for any elective destructive procedures. Informed consent means that the patient must fully understand that alternative safer or more biologically sound treatment options actually do exist for them.

### 3.4. Management of Worn Incisors

Managing worn incisors using a conservative approach is a critical part of the overall approach to biologically sound TW management. Incisors are relatively small teeth, and therefore, what little structure they have left following significant wear needs to be protected and preserved.

**Figure 6:**
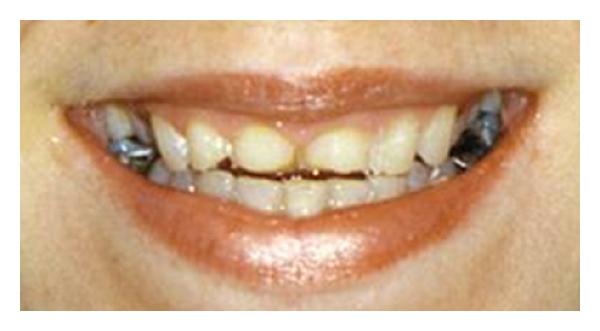
Photograph of a female patient with erosion caused by bulimia.

Figure 6 shows photographic images of a female patient with TW, resulting in short maxillary anterior clinical crown height. The aetiology was erosion from intrinsic acid as a result of bulimia, to which the patient readily admitted on direct questioning. She had previously been managed on a “watch and wait” basis by the making of impressions to provide stone casts of her teeth on a yearly basis for seven years. This approach proved futile and costly in terms of both valuable sound tooth tissue and expensive clinical time.

**Figure 7:**
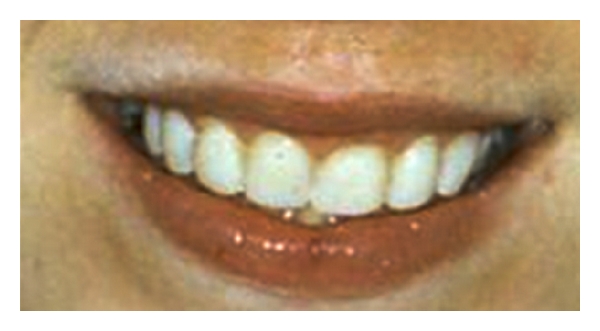
Photograph of reversible “mock up” with direct resin composite on the unetched enamel of just the eroded anterior teeth to allow patient evaluation prior to treatment. This “mock up” or composite simulation was done to check that it met with the patients approval of the proposed changes in appearance. Once it did, the temporary composite "mock up" was removed and just the anterior eroded teeth were directly bonded freehand at the agreed increased vertical dimension with hybrid resin composite.

A resin composite “mock up” on the unetched, but dried enamel was used to show the patient what could be done to change her dental appearance. As the patient liked that appearance, the temporary mock up resin composite was removed, the affected teeth were then etched, and a three bottle bonding system was used prior to placing resin composite freehand directly onto just the anterior eroded teeth, at an increased anterior occlusal vertical dimension. This was done without damaging the already eroded teeth in any way or involving the mainly intact posterior teeth. Pragmatic “one-hit” direct resin composite bonding has been shown to be clinically successful with minimal or no-long-term iatrogenic effects [29, 31–33], and the use of three-step (etch, primer, and bond) bonding system is still the gold standard for mixed enamel/dentine bonding [34].  Figure 7 shows the result after the affected teeth were temporarily “mocked up” with directly applied hybrid resin composite at an increased occlusal vertical dimension. This resulted in improved aesthetics with minimal biological costs, as the residual eroded tooth tissue was protected in one appointment without any treatment of the posterior dentition, which returned to full occlusal contact within 3 months. Increasing the anterior occlusion only is a predictable and safe procedure with a mean time to re-establishing posterior occlusal contacts of 7 months [29, 31]. It should be explained to patients to be treated with this approach that they will need time to adapt to the changes in their occlusion. The resin composite acts as a direct fixed orthodontic device and the teeth are protected by proprioception in the periodontal ligaments while the patient adapts [31]. Confidence in this procedure is based on work originally undertaken by Anderson in 1962 showing that patients readily adapt to changes in their occlusion [32–35]. These original studies have now been backed up by 30 years of follow-up data showing minimal or no-long-term biological costs occur from this increase in the occlusal vertical dimension [36].

**Figure 8:**
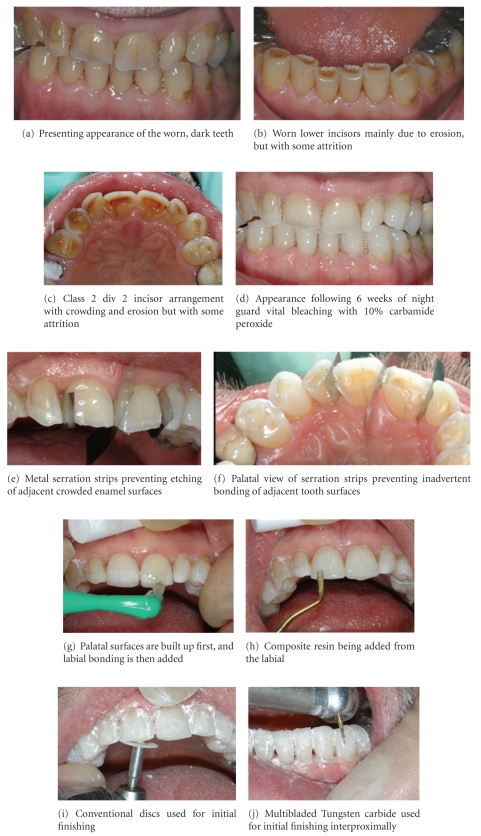

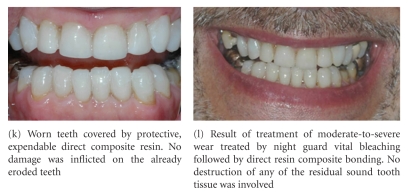
Patient showing moderate-to-severe wear treated by night guard vital bleaching followed by direct resin composite bonding. No destruction of any of the residual sound tooth tissue was involved.

Figure 8 shows some of the clinical procedures used to treat a patient using this approach.

### 3.5. Management of Worn Molars

**Figure 9:**
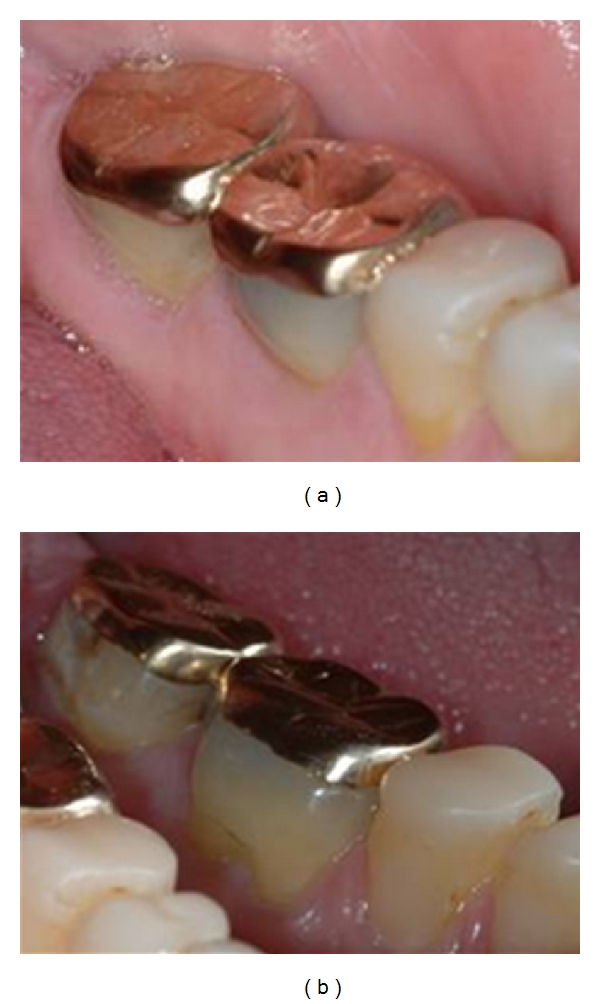
Management of posterior TW using adhesively retained gold onlays at 15 years after cementation.

Management of generalised TW can be facilitated by raising the occlusal vertical dimension using direct composite resin bonding techniques in the incisor and canine areas, thus opening the posterior occlusion. If the TW is generalised, this space can be secured by temporarily placing fast setting, contrasting colour, glass ionomer cement onto the molar teeth at the increased anterior vertical dimension. These provisional restorations will secure the space gained by the anterior bonding. The premolars or molars in between these newly bonded teeth can then be definitively restored at leisure during subsequent appointments with an appropriate approach and materials, all depending on the degree of wear or the existing restorations in the posterior teeth.  Figure 9 shows photographs of gold adhesive onlays used to restore worn posterior teeth at 15 year followup [37].

If the premolar or molar teeth have existing large restorations and need conventional crowns or onlays, then the longer preparations that are made possible, due to the fact that no further occlusal reduction is required, mean that these longer castings made possible in this biologically sensible way will be better retained with either conventional or resin-based cements as indicated.

### 3.6. Where Now with Wear?

These biologically based approaches advocated in this paper are worthy of equal, if not greater, financial reward than traditional destructive approaches using conventional crown and bridgework. Dentists who are able to practice and hone their skills using additive techniques with resin composite and not subtractive techniques with a dental bur usually have greater artistic craft skills and a profounder appreciation of the biologic long term value of their patients remaining sound enamel and dentine.

These valuable skills are more likely to be sought after by sensible patients with TW, and when they understand the real issues they will usually be willing to pay a fair fee for this biologically sound approach which ensures that they retain their remaining sound tooth tissue with their intact and healthy pulps. Only once minimally destructive approaches have been explored and exhausted should clinicians consider the “medium destructive” approaches and only then consider the highly destructive tooth preparations required for full mouth rehabilitations.

In the authors' view, the philosophy of full mouth reconstruction using conventional fixed prosthodontics has very limited biological advantages. The preparation of multiple sound or minimally worn teeth for indirect all-porcelain or porcelain-fused-to-metal restorations is not justifiable, simply because teeth elsewhere in the dentition have been affected by TW, or because it is easier, traditional, or more lucrative, for the clinician to aggressively treat the TW in this manner.

Regretfully, many remuneration systems and prosthodontic training courses have retained an emphasis on teaching highly destructive management strategies which have a very poor fallback position (i.e., the ability to repair, retrieve, and retain the teeth and place new sound restorations) when problems inevitably occur.

## 4. Conclusion

This paper has argued that a paradigm shift is now required in relation to managing tooth wear. For far too long, the emphasis in managing tooth wear has been on the wrong aspects, namely, the length of time that the dental restoration is successful or “survives”. The emphasis has to change to a more biologically sensible, patient-centred approach, involving minimal destruction of their worn teeth and an acceptance that the materials used to repair or protect the worn tooth tissue will need to be repaired, repolished, renewed, or recycled as required. Damage or chipping of the resin composite itself is of minimal consequence, as it can be readily repaired, whereas the residual load-bearing sound tooth structure and pulpal health are invaluable.

The focus for restorative management of tooth wear should, therefore, be on using additive, constructive prosthodontic skills rather than relying on the destructive approaches and skills involved in the traditional full mouth reconstruction using full-ceramic or cast-ceramic veneered restorations. In biologic terms, the destruction of sound tooth structure or the hazarding of dental pulps can no longer be condoned by sensible, caring dentists, whose trusting patients depend on when seeking help with their tooth wear.
